# Public health policies and interventions to address health inequities in high-income countries: an umbrella review

**DOI:** 10.1186/s12889-025-25876-2

**Published:** 2026-02-05

**Authors:** Jatinder Hayre, Ellie Canning, Helen Pearce, Raj Khera, John Ford

**Affiliations:** 1 Wolfson Institute of Population Health, Queen Mary University of London, London, UK; 2 Norfolk and Norwich University Hospital, Norwich, UK; 3 St. Georges University of London, London, UK

**Keywords:** Health inequities, Social determinants of health, Public health policy, Umbrella review, Redistributive interventions, Legislative measures, Housing and community interventions, Health system interventions, Telehealth, Intervention-Generated inequality

## Abstract

**Background:**

Health inequity: defined as systematic and avoidable difference in health outcome, remain entrenched across high-income countries, with socioeconomic gaps in life expectancy exceeding 7–10 years. Upstream interventions addressing the social determinants of health are critical. This umbrella review evaluates which macro-level policies and public health interventions most effectively reduce health inequity.

**Methods:**

We conducted an umbrella review of systematic reviews. Four databases (Embase, Medline, Scopus, Cochrane) were searched from May 2017, the date of the last umbrella review on the subject, to September 2024. Eligible reviews reported population-level interventions in OECD countries, with outcomes stratified by socioeconomic status or related disadvantage. Included systematic reviews were appraised using AMSTAR II. We devised a conceptual Health Equity Pyramid that classified interventions by their agentic demand and population reach.

**Results:**

Thirty-five systematic reviews were included. This review evaluated evidence across six policy domains. Redistributive and welfare interventions, including cash transfers, basic income and food subsidies, consistently improved food security, household financial stability and maternal–child health outcomes. Legislative and regulatory measures, such as smoke-free policies and pharmaceutical subsidy reforms, demonstrated robust population-level gains, particularly in disadvantaged groups. Community and housing interventions improved psychological health, reduced morbidity and mortality in targeted populations, and enhanced housing stability. Health system interventions, notably tailored smoking cessation and hospital discharge coordination for people experiencing homelessness, were effective in narrowing disparities. By contrast, educational and behavioural programmes and telehealth interventions often demanded high individual agency; without contextual tailoring, may exacerbate intervention-generated inequality.

**Conclusions:**

This umbrella review demonstrates that interventions characterised by low agentic demand: welfare reform, housing support, and legislative measures; yield the most consistent reductions in inequity. High agentic interventions can be effective when carefully tailored to disadvantaged populations but may otherwise exacerbate disparities. Future policy should prioritise structural, population-level strategies to achieve sustainable equity in health outcomes.

**Trial registration:**

CRD42024529176.

**Supplementary Information:**

The online version contains supplementary material available at 10.1186/s12889-025-25876-2.

## Introduction

Health inequity, defined as systematic differences in health that are socially produced, unfair, and avoidable, remains an urgent concern across high-income nations [1, 2]. These inequities largely originate from the Social Determinants of Health (SDoH) [3]. Despite significant biomedical advances, the gap in life expectancy between socioeconomically disadvantaged communities and their more affluent counterparts can surpass 7 years; extending beyond 10 years, in certain high-income countries [4]. Moreso, the COVID-19 pandemic has underscored the fragility of health equity across multiple regions: demonstrating, across 17 European countries, excess mortality rates during peak waves were consistently 40–50% higher in socioeconomically deprived urban areas compared with national averages [5]. A parallel analysis in the United States, which examined over 3,000 counties, found that lower-income regions recorded nearly double the pandemic-related mortality of more affluent counties [6]. Such stark inequities are not mere coincidence; reflecting entrenched social disadvantage in SDoH: limited healthcare provision, precarious employment, and suboptimal housing; conditions that amplify the risk of preventable illness [7, 8].

The socio-political burden of health inequity is stark. Health inequities exert a substantial economic burden in high-income countries, across the European Union, this translates to into a welfare loss approaching €980 billion annually: 9.4 per cent of total EU GDP [9]. In the US, the cumulative economic burden of health inequity totals $1.429 trillion [10]. Economic burden arises through complex multi-dimensional pathways. This includes direct healthcare expenditure through excess rates of emergency hospitalisation, preventable readmissions, and multimorbidity treatment costs [11]; reduced labour market participation and lower overall productivity amongst disadvantaged populations [12]; and, through inequitable educational outcomes and subsequent limited employment opportunities perpetuating lower earnings and reduced tax revenues [13]. Fiscal analyses highlight how directed investments in upstream SDoH interventions, including progressive welfare schemes and improved housing infrastructures, can substantially reduce the financial burden on health systems and yield sustained benefits for disadvantaged groups [14, 15]. Nonetheless, there has been a paradigm shift in public health discourse towards interventions focussing on individual agency and framing the narrative towards personal accountability [16, 17].

Since the last umbrella review on the subject by Thomson et al. from 2018 [18], which itself extended the earlier work by Lorenc (2013) [19] and the seminal work on the subject by Bambra (2010) [20] there has been a rapid expansion of the evidence base following a significant increase and renewed focus on health equity research after the COVID-19 pandemic [21]. Whilst health disparities have widened following the COVID-19 pandemic [22], there has been an increase in published studies focussing on interventional research on health equity [23], though parity with descriptive studies is distant. Therefore, this review is both timely and urgently warranted.

Within this review, public health interventions were defined as: *activities or programmes that promote or protect health*,* or prevent ill-health in populations*,* encompassing policy measures*,* service provision*,* environmental change*,* and health promotion* [24]. Interventions were conceptualised through the Health Equity Pyramid as operating along a gradient of individual agency and population impact, with structural and systemic actions at the base exerting the greatest potential to reduce health inequalities.

This umbrella review aims to determine which specific interventions and macro-level policy strategies effectively address health inequity and the SDoH, through a robust systematic synthesis of both quantitative and qualitative evidence. In so doing, the review will identify the most appropriate strategies to address health inequity, possible evidence gaps, outline future research priorities, and offer evidence-based insights for policymakers, healthcare leaders, and stakeholders committed to fostering equitable, resilient societies at scale.

## Methods

### Conceptual framework

**Fig. 1:**
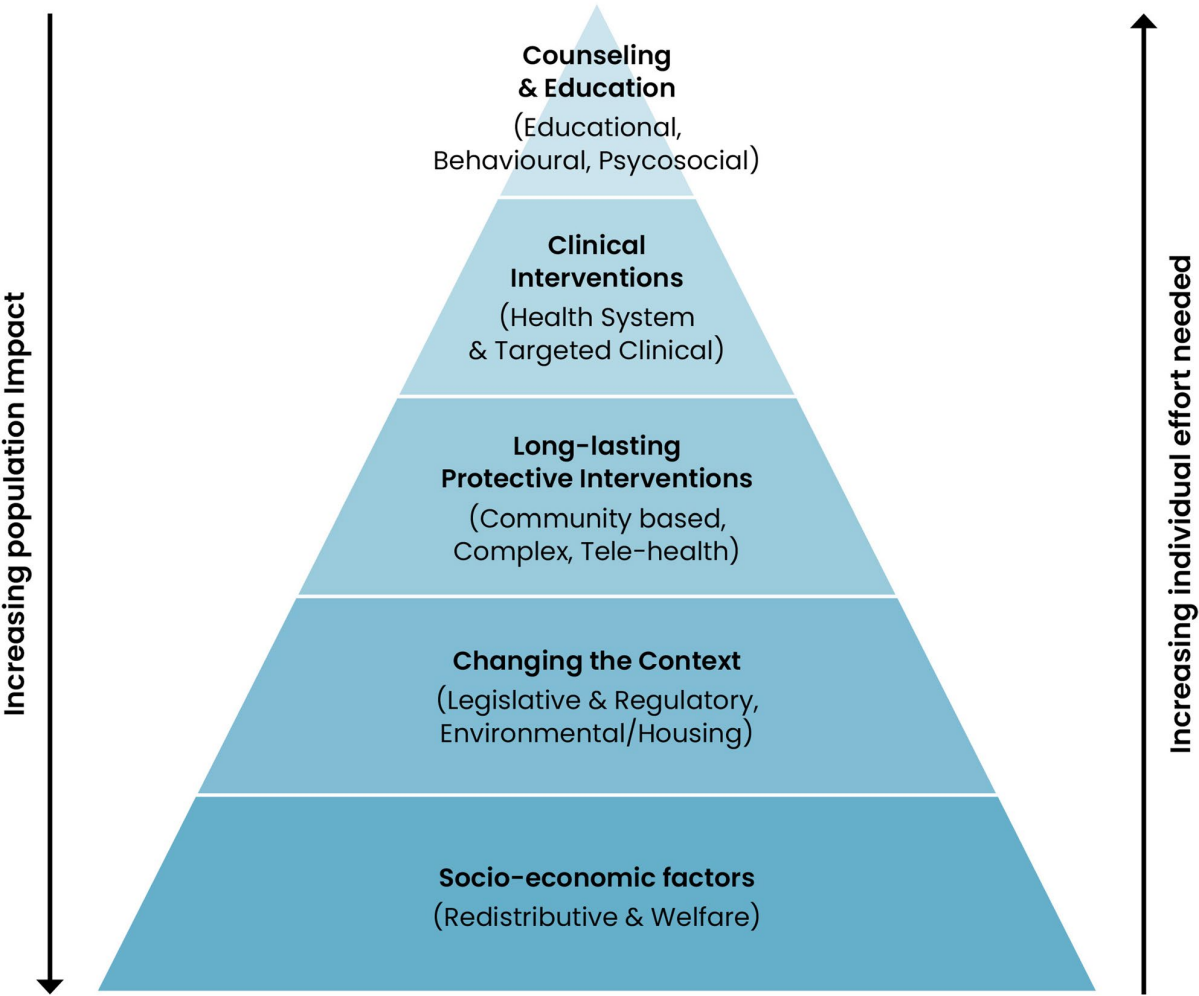
Health equity pyramid

This review was guided by a priori conceptual framework; we adapted Frieden’s Health Impact Pyramid [25] into a five-tier *Health Equity Pyramid* to guide narrative synthesis of the included systematic review studies: this is illustrated as Fig. 1: Health Equity Pyramid. The adapted model orders interventions along a continuum of required individual agency versus anticipated population reach: (i) redistributive and welfare policies that reshape socioeconomic factors; (ii) legislative and regulatory measures that alter default environmental conditions; (iii) long-lasting protective interventions, such as multi-component and community-based programmes; (iv) clinical interventions embedded in the health system; (v) educational, behavioural, and psychosocial programmes demanding sustained personal engagement. This classification facilitated aggregation of heterogeneous evidence but compounded the equity implications inherent in differing degrees of agentic demand, thereby enabling systematic comparison of intervention classes within and across domains. Within this framework, the Health Equity Pyramid offers a five-tier conceptual structure rather than a set of analytical categories that map in a strict one-to-one fashion onto the results. Telehealth and remote approaches are situated within the same conceptual layer as complex protective interventions in Fig. 1, yet the evidence demonstrated distinctive delivery patterns and equity mechanisms that justified discrete analysis. The results therefore comprise six domains that remain grounded in the five overarching conceptual tiers, with telehealth presented as a differentiated expression of this shared conceptual space.

### Design

We utilised an umbrella review methodology: a structured review of systematic reviews.

### Search strategy

We undertook a systematic search of four databases: Embase, Medline, Scopus, and Cochrane in September 2024 (search terms shown in Supplementary appendix 1). The search terms were adapted to each specific database search engine. The search was limited to publication date after May 2017; when the previous umbrella review on the subject ran the last search [18].

### Inclusion and exclusion criteria

**Fig. 2:**
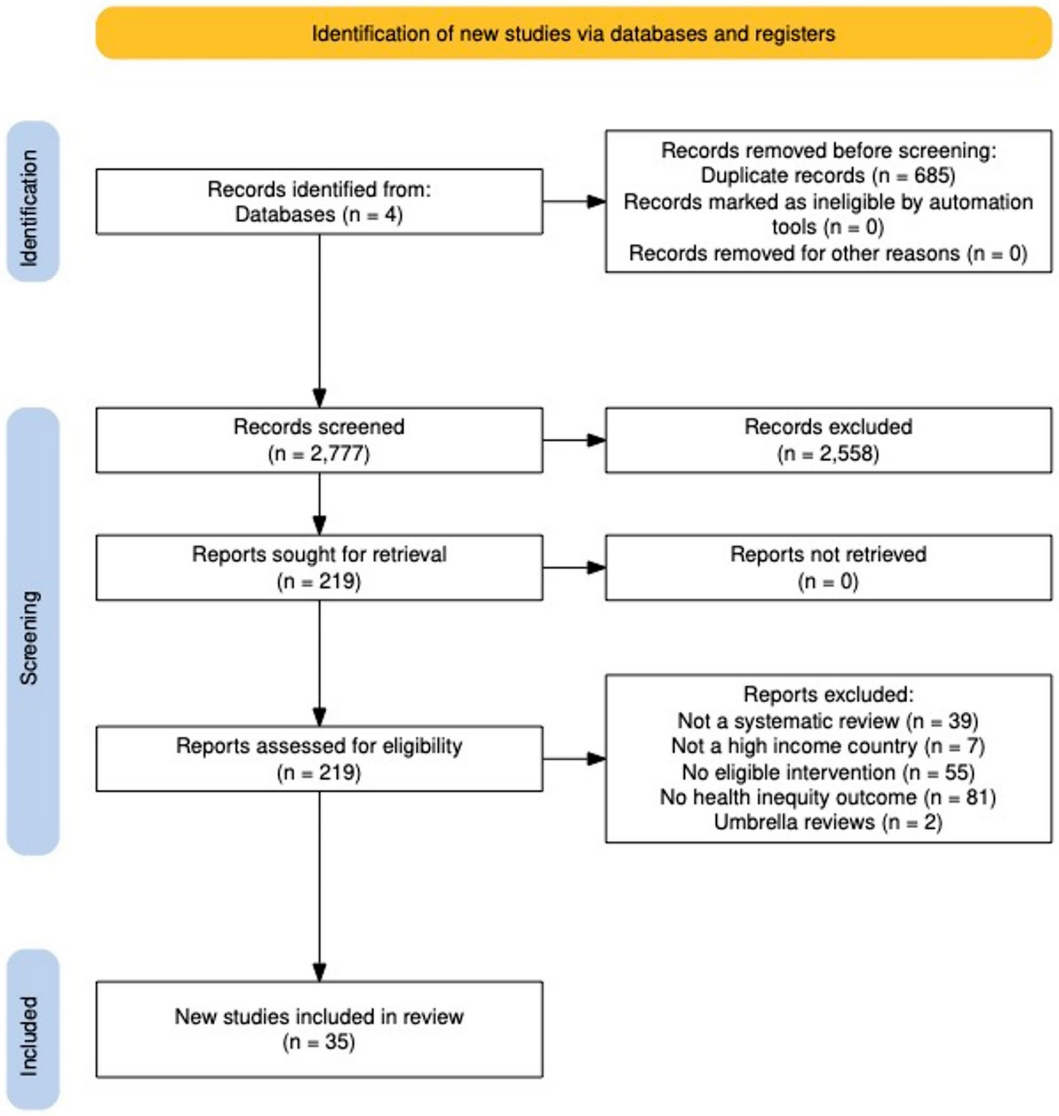
PRISMA flow diagram

The eligibility criteria were established a priori, employing the PICOS (population, intervention, comparison, outcomes, setting) framework:

- *Population*: Children and adults of any age residing in high-income countries, defined as member states of the OECD.
- *Intervention*: Population-level public-health policies and interventions implemented at national, regional, or municipal scale. Classified as either high agentic or low agentic demand; as per the conceptual framework (health impact pyramid): interventions situated at the lowest two tiers, near the base, are classified as low agentic demand; those within the upper two tiers reflect high agentic demand, and the intermediate tier denotes moderate agentic demand.
- *Comparison*: Systematic reviews were admissible irrespective of the underlying studies’ employed control groups.
- *Outcomes*: The primary outcomes measures were health inequity by socioeconomic status (SES) defined as: income, wealth, education, employment, occupational class, welfare-receipt or area-level deprivation; and, where relevant, ethnicity but only in the explicit context of low-SES.
- *Study Design*: Systematic reviews of quantitative and qualitative interventional studies. Consistent with prior umbrella methodologies, each publication had to satisfy the two mandatory Database of Abstracts of Reviews of Effects (DARE) criteria [26]: (i) a clearly articulated review question specifying at least two PICOS elements, and (ii) a structured search that interrogated a minimum of one named bibliographic database augmented by reference checking, hand searching, citation tracking, or author contact. This is outlined in Fig. 2: the PRISMA flow diagram.

### Study selection

All retried records were imported into EndNote reference manager, for deduplication. Titles and abstracts were first double screened by two reviewers (JH, EC) against the pre-specified eligibility criteria. All records meeting those criteria proceeded to full-text scrutiny by the same authors, with an additional 10% random sample check by a third reviewer (RK). Core data was then extracted by the lead author (JH) and independently verified for completeness and accuracy by a second reviewer (EC). Discrepancies which arose were resolved through meetings between the lead author (JH) and second reviewer (EC). Reasons for exclusion at the full-text stage were documented and are included in the PRISMA flow diagram. The study selection process adhered to PRISMA 2020 guidelines.

### Data extraction

A purpose-built spreadsheet captured standardised data, namely: bibliographic details; participant descriptors; study design; intervention characteristics; principal findings, defined as key reported results which directly address this umbrella review’s research question; outcome metrics; methodological strengths, weaknesses, and the overall quality rating, utilising the AMSTAR II tool. The full extracted dataset was comprehensively verified by two of the reviewers (JH, RK). The aim was for each systematic review to be mapped to the highest tier that captured its principal mechanism of effect; discrepancies were resolved through consensus adjudication. Additionally, this umbrella review utilised the PROGRESS-Plus framework: a recognised tool for applying an equity lens on interventional evidence and assessing differential outcomes in disadvantaged populations who traditionally remain underrepresented in research [27, 28]. Each included review was assessed for whether these characteristics were reported. Additional “Plus” variables, migration status and medical insurance coverage, were recorded descriptively to reflect the range of equity considerations represented within the evidence base.

### Data synthesis

Data synthesis relied on information reported in each systematic review, including any supplementary files. Results were then analysed and correlated to the Health Equity Pyramid conceptual framework and integrated into a narrative synthesis. Six policy domains were devised through deliberation between two researchers (JH, JF). We characterised three archetypal themes, capturing the six policy domains: (1) low agentic demand-high population impact studies; (2) high agentic demand-low population impact studies; (3) Intervention-generated inequalities.

## Results

### Search outcome

This umbrella review builds upon Thomson et al. from 2018 [18], extending the evidence base from May 2017 onwards, marking the end of the previous review’s search period. The updated search identified 35 new systematic reviews; exclusively in high-income countries, published since the prior review, reflecting the expansion of evidence. Searches across four electronic databases: MEDLINE, Embase, Scopus, and Cochrane, yielded 2,777 unique records following the removal of 685 duplicates. After title and abstract screening, 2,558 records were excluded as not meeting eligibility criteria. A total of 219 full-text articles were subsequently assessed for inclusion, of which 184 were excluded: 39 were not systematic reviews, 7 were conducted in non-high-income countries, 55 did not include an eligible intervention, 81 did not report inequity-related outcomes, and 2 were umbrella reviews. The study selection process is illustrated in Fig. 2: PRISMA flow diagram, outlining the stages of identification, screening, eligibility assessment, and inclusion.

### Statement of principal findings

The included studies delineate into six distinct domains, each comprising public health interventions or policy strategies targeting disadvantaged populations. The includes systematic reviews are included in tables 3–8: categorised according to policy domain. These six policy domains were situated within the five-tier Health Equity Pyramid, a conceptual framework that orders interventions according to their anticipated population reach and the degree of individual effort required to benefit. Within the redistributive and welfare domain, interventions including conditional cash transfers, food prescription programmes and basic income pilots consistently enhanced short-term outcomes related to food security, household financial stability and subjective well-being. Educational, behavioural and psychosocial interventions produced mixed results depending on precise intervention type; parent-focused weight management evidenced modest improvements in health-proxy behaviours such as health literacy, dietary behaviours and shift around smoking; yet, clinical outcomes and endpoints were not included, and variability in intervention fidelity compromised the consistency of findings. Moreso, multiple interventions within this domain: workplace interventions, school-based programmes, self-management, also evidenced comparatively poorer outcomes in low-SES populations compared to higher-SES groups. In the domain of community and housing interventions, multicomponent housing support initiatives and tenant-based voucher programmes provided robust evidence of reduced housing instability, improved psychological health, reduced morbidity and mortality in certain disease subgroups, and early cardiometabolic benefits. Health-system interventions demonstrated encouraging results regarding service utilisation, accessibility, and engagement in low-income populations with high-service needs. Legislative and regulatory policies, such as smoke-free legislation and structural insurance reform, demonstrated clear population-level health improvements. Telehealth and digital interventions provided initial evidence of improved health behaviours and engagement for disadvantaged communities, particularly when linguistic and technological barriers were explicitly addressed; however, evidence for sustained health improvements and equitable distribution remains preliminary.

### Participant characteristics: PROGRESS-Plus mapping

Of 35 systematic reviews, 34 reported income. Only four reviews examined “Plus” variables, specifically migrant status and medically uninsured status. The pattern indicates near-universal attention to socioeconomic status, with comparatively sparse attention to other modalities of disadvantage. This is exemplified in Table 1: a summary of PROGRESS-Plus characteristics for each of the included studies.

**Table 1:** PROGRESS-plus characteristics summary table

Review (Citation)	Place of Residence	Race/Ethnicity	Occupation	Gender/Sex	Religion	Education	Socio‑economic Status	Social Network/Capital	Plus
Fuller et al. 2022 [29]	**–**	**–**	**–**	**–**	**–**	**–**	**+**	**–**	**–**
Boccia et al. 2021	**–**	**–**	**–**	**–**	**–**	**–**	**+**	**–**	**–**
Haslam et al. 2022 [30]	**–**	**–**	**–**	**–**	**–**	**–**	**+**	**–**	**–**
McKay et al. 2020	**+**	**–**	**–**	**–**	**–**	**–**	**+**	**–**	**–**
Haslam et al. 2022 [30]	**–**	**–**	**–**	**–**	**–**	**–**	**+**	**–**	**–**
Gibson et al. 2019	**–**	**–**	**+**	**–**	**–**	**–**	**+**	**+**	**–**
Troy et al. 2022 [31]	**–**	**–**	**–**	**–**	**–**	**–**	**+**	**–**	**–**
Littlecott et al. 2022 [32]	**+**	**–**	**–**	**–**	**–**	**–**	**○**	**–**	**–**
McDarby and Looney 2023	**–**	**–**	**–**	**–**	**–**	**–**	**+**	**–**	**–**
Orciari et al. 2022 [33]	**+**	**–**	**–**	**–**	**–**	**–**	**+**	**–**	**–**
Hardman et al. 2020 [34]	**–**	**–**	**–**	**–**	**–**	**–**	**+**	**–**	**–**
Mathiesen et al. 2019 [35]	**–**	**+**	**–**	**–**	**–**	**–**	**+**	**–**	**–**
Robinson et al. 2023 [36]	**–**	**–**	**–**	**–**	**–**	**–**	**+**	**–**	**–**
Garbers et al. 2018 [37]	**–**	**+**	**–**	**–**	**–**	**+**	**+**	**–**	**–**
Feteira-Santos et al. 2020 [38]	**–**	**–**	**–**	**–**	**–**	**–**	**+**	**–**	**–**
Shreshta et al. 2023	**–**	**–**	**–**	**–**	**–**	**–**	**+**	**–**	**–**
McGrath et al. 2022	**+**	**–**	**–**	**–**	**–**	**–**	**+**	**–**	**–**
Nickel and Von Dem Knesebeck 2020 [39]	**+**	**–**	**–**	**–**	**–**	**–**	**+**	**–**	**–**
Onapa et al. 2022 [40]	**+**	**–**	**–**	**–**	**–**	**–**	**+**	**–**	**–**
Slopen et al. 2019	**+**	**–**	**–**	**–**	**–**	**–**	**+**	**–**	**–**
Finnie et al. 2022 [41]	**+**	**–**	**–**	**–**	**–**	**–**	**+**	**–**	**–**
Ellis et al. 2022 [42]	**–**	**+**	**–**	**–**	**–**	**–**	**+**	**–**	**–**
Raison and Harris 2019 [43]	**–**	**–**	**–**	**+**	**–**	**–**	**+**	**–**	**–**
Anyiwe et al. 2024 [44]	**–**	**–**	**–**	**+**	**–**	**–**	**+**	**–**	**+**
Allen et al. 2021	**+**	**–**	**–**	**–**	**–**	**–**	**+**	**–**	**–**
Smith et al. 2020 [45]	**+**	**–**	**–**	**–**	**–**	**–**	**+**	**–**	**–**
Luchenski et al. 2022 [46]	**+**	**–**	**–**	**–**	**–**	**–**	**+**	**–**	**–**
Nanninga et al. 2019 [47]	**–**	**–**	**–**	**–**	**–**	**–**	**+**	**–**	**+**
Sanchez-Vaznaugh et al. 2023	**–**	**+**	**–**	**+**	**–**	**+**	**+**	**–**	**–**
Kaplan et al. 2024 [48]	**–**	**+**	**–**	**–**	**–**	**–**	**+**	**–**	**+**
Guindon et al. 2022 [49]	**–**	**–**	**–**	**–**	**–**	**–**	**+**	**–**	**+**
Western et al. 2023	**–**	**–**	**–**	**–**	**–**	**–**	**+**	**–**	**–**
Katz et al. 2023	**+**	**–**	**–**	**–**	**–**	**–**	**+**	**–**	**–**
Obregon et al. 2024 [50]	**–**	**–**	**–**	**–**	**–**	**–**	**+**	**–**	**–**
Egan et al. 2023	**+**	**–**	**–**	**–**	**–**	**–**	**+**	**+**	**–**

Key: “+” for explicitly analysed domains

“–” for no mention

“○” for included but not analysed

### Critical appraisal

Using AMSTAR II across sixteen methodological domains [51], 35 systematic reviews were appraised: three satisfied all critical domains and were rated high; sixteen missed a single non-critical element and was rated moderate; thirteen had a single critical-domain deficit and were rated low; only three presented two or more critical deficits and were rated critically low. The most recurrent weaknesses related to the absence of preregistered protocols, incomplete or narrowly justified search strategies, and the omission of publication-bias assessment in meta-analyses. A smaller number of reviews did not sufficiently integrate study quality into their interpretation of findings This is summarised in Table 2: a summary of AMSTAR II ratings across domains for each included study (Tables 3, 4, 5, 6, 7, and 8).

**Table 2:** AMSTAR II Ratings Summary of Included Studies

Study	Item1	Item2	Item3	Item4	Item5	Item6	Item7	Item8	Item9	Item10	Item11	Item12	Item13	Item14	Item15	Item16	Overall
Egan et al. 2024 [52]	Y	Y	PY	PY	N	PY	Y	Y	Y	N	NA	Y	Y	Y	NA	Y	Moderate
Onapa et al. 2022 [40]	Y	PY	Y	PY	N	PY	Y	Y	Y	N	NA	Y	Y	Y	NA	Y	Moderate
Orciari et al. 2022 [33]	Y	Y	Y	PY	N	PY	Y	Y	Y	N	NA	Y	Y	Y	NA	Y	Moderate
Ellis et al. 2022 [42]	Y	Y	Y	PY	PY	PY	Y	Y	Y	N	NA	Y	Y	Y	NA	Y	Moderate
Luchenski et al. 2022 [46]	Y	Y	Y	PY	PY	PY	Y	Y	Y	N	NA	Y	Y	Y	NA	Y	Moderate
Western et al. 2021 [53]	Y	N	Y	PY	Y	Y	Y	Y	Y	N	Y	Y	Y	Y	Y	Y	Low
McGrath et al. 2021 [54]	Y	Y	Y	Y	Y	Y	Y	Y	Y	N	NA	Y	Y	Y	NA	Y	Moderate
Nickel & Von Dem Knesebeck, 2020 [39]	Y	N	Y	PY	N	N	Y	Y	Y	N	NA	Y	Y	Y	NA	Y	Low
Hardman et al. 2020 [34]	Y	Y	Y	PY	PY	N	Y	Y	Y	N	NA	Y	Y	Y	NA	Y	Moderate
Allen et al. 2020 [55]	Y	Y	Y	Y	Y	Y	Y	Y	Y	N	NA	Y	Y	Y	NA	Y	Moderate
Smith et al. 2020 [45]	Y	N	Y	Y	PY	PY	Y	Y	Y	N	NA	Y	Y	Y	NA	Y	Moderate
Mathiesen et al. 2019 [35]	Y	N	Y	PY	Y	Y	Y	Y	Y	N	Y	Y	Y	Y	Y	Y	Low
Nanninga et al. 2018	Y	Y	Y	PY	PY	PY	Y	Y	Y	N	Y	Y	Y	Y	Y	Y	Moderate
Slopen et al. 2018 [56]	Y	N	Y	PY	Y	PY	Y	Y	Y	N	NA	Y	Y	Y	NA	Y	Low
Gibson et al. 2018	Y	Y	Y	Y	Y	Y	Y	Y	Y	N	Y	Y	Y	Y	Y	Y	High
Boccia et al. 2023 [57]	Y	Y	Y	Y	Y	PY	Y	Y	Y	N	NA	Y	Y	Y	NA	Y	Moderate
Sanchez-Vaznaugh et al. 2023	Y	N	Y	PY	Y	PY	Y	Y	Y	N	NA	Y	Y	Y	NA	Y	Critically Low
Katz et al. 2024 [58]	Y	Y	Y	Y	Y	Y	Y	Y	Y	N	Y	Y	Y	Y	Y	Y	High
Finnie et al. 2022 [41]	Y	N	Y	PY	Y	Y	Y	Y	Y	N	NA	Y	Y	Y	NA	Y	Low
McKay et al. 2023 [59]	Y	N	Y	PY	Y	Y	Y	Y	Y	N	NA	PY	Y	Y	NA	Y	Low
Troy et al. 2022 [31]	Y	Y	Y	PY	PY	PY	Y	Y	Y	N	NA	Y	Y	Y	NA	Y	Moderate
van Heijster et al. 2021 [60]	Y	Y	Y	Y	Y	PY	Y	Y	Y	N	Y	N	Y	Y	N	Y	Low
Fuller et al. 2022 [29]	Y	Y	Y	Y	Y	PY	Y	Y	Y	N	NA	Y	Y	Y	NA	Y	Moderate
Shrestha et al. 2023 [61]	Y	Y	Y	PY	PY	PY	Y	Y	Y	N	NA	PY	Y	Y	NA	Y	Moderate
Robinson et al. 2023 [36]	Y	Y	Y	Y	Y	Y	Y	Y	Y	N	Y	Y	Y	Y	Y	Y	High
Raison & Harris et al. 2019 [43]	Y	N	Y	PY	Y	Y	Y	Y	Y	N	NA	Y	Y	Y	NA	Y	Low
Anyiwe et al. 2024 [44]	Y	Y	Y	PY	Y	Y	Y	Y	Y	N	NA	PY	Y	Y	NA	Y	Moderate
Feteira-Santos et al. 2020 [38]	Y	N	Y	PY	Y	Y	Y	Y	Y	N	NA	PY	Y	Y	NA	Y	Low
Guindon et al. 2023	Y	Y	Y	Y	Y	Y	Y	Y	Y	N	NA	N	PY	Y	N	Y	Low
McDarby & Looney, 2024 [62]	Y	N	PY	Y	N	PY	Y	Y	Y	N	NA	PY	Y	Y	N	Y	Critically Low
Obregon et al. 2024 [50]	Y	N	Y	PY	Y	Y	Y	Y	Y	N	NA	PY	Y	Y	NA	Y	Low
Haslam et al. 2022 [30]	Y	N	Y	PY	PY	Y	Y	Y	Y	N	Y	PY	Y	Y	Y	Y	Low
Garbers et al. 2018 [37]	Y	N	PY	PY	N	PY	Y	Y	N	N	NA	NA	N	Y	NA	Y	Critically Low
Kaplan et al. 2024 [48]	Y	Y	Y	PY	Y	PY	Y	Y	N	N	NA	NA	N	Y	NA	Y	Low
Littlecott et al. 2022 [32]	Y	Y	Y	PY	Y	PY	Y	Y	Y	N	NA	Y	Y	Y	NA	Y	Moderate

**Table 3:** Summary of included reviews reporting redistributive and welfare interventions

Study	Population	Intervention(s)	Outcomes	Number of Primary Studies	Summary of results	AMSTAR II quality appraisal
Fuller et al. 2022 [29]	Low-income children aged 0–18 and their parents, based in Canada.	Cash transfer welfare programmes (conditional and unconditional).	Child development, parental mental health, household food security, healthcare engagement.	23	Conditional and unconditional cash transfers modestly boosted early cognitive and behavioural development in children, improved parental anxiety and depression, and increased healthcare use; child-health endpoints inconsistent, no equity stratification.	Moderate
Boccia et al. 2021	Low-income households with children across high-income countries.	Unconditional and conditional cash transfers.	Financial security, parental stress, child development.	30	Income-support programmes yielded mixed findings; school attendance, food security, and care access improved most for early-life recipients, but overall pattern varied, and equity impact remained unclear.	Moderate
Haslam et al. 2022 [30]	Low-income populations with a diet related chronic disease (e.g. obesity, diabetes, hypertension, or cardiovascular disease)	Food pharmacy programme intervention.	Four main outcomes: hemoglobin A1c, body mass index (BMI), daily fruit and vegetable servings, and systolic blood pressure.	17	Food-prescription schemes raised fruit-and-vegetable intake by 0.77 servings day⁻¹ (95 % CI 0.30 to 1.24) and lowered BMI by 0.40 kg m⁻² (95 % CI −0.50 to −0.31); no significant change in HbA1c or systolic blood pressure, evidence mainly non-randomised.	Low
McKay et al. 2020	Children and families in urban areas.	Basic income pilot schemes (e.g. MINCOME).	Hospitalisation rates, psychological stress, well-being.	10	Basic-income pilots reduced hospitalisations, enhanced subjective wellbeing, and lowered stress in low-income adults; lack of subgroup analyses leaves equity effects uncertain.	Low
Gibson et al. 2019	Lone parents on welfare in OECD countries.	Welfare-to-work schemes.	Depression, self-rated health, employment.	12	Short-term mental health benefits (defined as anxiety, depression, stress, overall well-being scores) noted post-intervention, particularly among women. Long-term health outcomes were lacking, and few studies examined differential effects by SES or ethnicity.	High
McGrath et al. 2022	Adults in socioeconomically deprived communities accessing co-located services.	Community-based welfare and advice services integrated within hubs.	Mental health, service uptake, financial security.	15	Community welfare, navigation and labour-market schemes for financially strained adults yielded uneven health gains; wellbeing rose only when finances improved (notably for women and Black participants); link-worker prescribing cut anxiety and service use; debt advice null; food banks helped food security and mental health; labour programmes lowered depression chiefly among higher-educated seekers.	Moderate

**Table 4:** Summary of included reviews reporting legislative and regulatory interventions

Study	Population	Intervention(s)	Outcomes	Number of Primary Studies	Summary of results	AMSTAR II quality appraisal
Nanninga et al. 2019 [47]	Children impacted by smoking bans	Public smoking bans	Exposure to second-hand smoke in the home	15	Smoke-free legislation cut child second-hand-smoke exposure by 28 % (RR 0.72; 95 % CI 0.62–0.83); heterogeneity extreme; one study showed 39 % cotinine fall but most lacked SES stratification.	Moderate
Sanchez-Vaznaugh et al. 2023	School-aged children; sub-groups by free-/reduced-price lunch (FRPL), gender, grade, race/ethnicity, SES	Competitive food-and-beverage policies that restrict or set standards for “competitive” snacks/drinks sold outside federal school-meal programmes	BMI percentile; prevalence of overweight/obesity	18	Competitive food-and-beverage policies produced mixed effects; stronger rules lowered BMI only in high-poverty Minnesota districts; no consistent differences by FRPL, gender, grade or race elsewhere.	Critically Low
Kaplan et al. 2024 [48]	Low-income and minority populations	Drug cost-sharing reforms, co-pay elimination	Medication adherence, pharmacy utilisation	19	Prescription-cost reforms (co-pay cuts, tiered subsidies, insurance expansion) reduced out-of-pocket spend and raised adherence, with largest gains for low-income and minoritised users; equity depended on eligibility thresholds and exemption design.	Low
Guindon et al. 2022 [49]	General health service users with low-income population	Public drug insurance	Medication access, unmet healthcare need	30	Public drug insurance lowered cost-related non-adherence and unmet need; several studies reported narrowed income and education gaps when reforms were paired with outreach and simplification, but disparities persisted where literacy and provider bias were unaddressed.	Low

**Table 5:** Summary of included reviews reporting community-based programmes, housing and complex multi-component interventions

Study	Population	Intervention(s)	Outcomes	Number of primary studies	Summary of results	AMSTAR II quality appraisal
Nickel and Von Dem Knesebeck 2020 [39]	Communities with social disadvantage across European and North American settings.	Community-based health promotion programmes.	General health, health behaviours, service utilisation.	23	Fifty-seven percent of 23 multicomponent neighbourhood programmes narrowed health or behavioural inequities; one showed mixed/adverse effects; two benefited only the most deprived or most exposed residents.	Low
Onapa et al. 2022 [40]	Adults experiencing homelessness	Housing provision	Mental wellbeing, housing stability	24	Permanent supportive housing for homeless people improved survival and immune status in those with HIV/AIDS and reduced depression or suicidal ideation in selected cohorts; other physical-health gains limited.	Moderate
Slopen et al. 2019	Low-income families with children	Housing assistance	Child health, residential stability	14	Housing vouchers stabilised residence and, in quasi-experimental/observational studies, improved child growth, asthma, behaviour and activity, though effects were outcome-specific and not universal.	Low
Finnie et al. 2022 [41]	Low-income renters in the US	Housing Choice Voucher programme	Housing quality, neighbourhood opportunity	27	Housing Choice Vouchers moved families to better, safer areas; small gains in employment, insurance and food security; fewer adult physical/mental health issues and childhood asthma; long-term earnings benefits for children who moved before age 13.	Low
Ellis et al. 2022 [42]	Low-income and ethnic minority families with infants	Safer sleep education and home visits	Adherence to safe sleep guidance	23	Home-based safer-sleep education increased adherence among high-risk families, with higher uptake in low-income and ethnically minoritised groups.	Low
Raison and Harris 2019 [43]	Disadvantaged pregnant women	Antenatal oral health education	Dental utilisation	6	Antenatal oral-health education with free dental packs boosted service uptake in disadvantaged pregnant women.	Moderate
Anyiwe et al. 2024 [44]	Migrants and people experiencing homelessness	Hepatitis B outreach and navigation	Screening, linkage to care	66	Outreach, culturally tailored messaging and navigation improved hepatitis B screening, knowledge and care linkage in migrants and homeless populations.	Moderate

**Table 6:** Summary of included reviews reporting health system interventions

Study	Population	Intervention(s)	Outcomes	Number of primary studies	Summary of results	AMSTAR II quality appraisal
Allen et al. 2021	Adults with non-communicable diseases in socioeconomically deprived communities.	Primary care and community-based chronic disease interventions.	Patient engagement, health literacy, chronic disease management.	17	Primary-care chronic-disease management and health-literacy support among disadvantaged adults increased patient activation, self-management and service engagement; evidence weak on closing health gaps.	Moderate
Smith et al. 2020 [45]	Socioeconomically deprived smokers across primary care and community settings.	Smoking cessation programmes delivered via primary care and outreach.	Quit rates, smoking prevalence.	27	Culturally tailored smoking-cessation services for low-SES groups raised quit rates in both brief advice and targeted programmes; likely contributes to narrowing tobacco inequities.	Moderate
Luchenski et al. 2022 [46]	People experiencing homelessness in hospital settings.	Integrated care coordination and discharge planning	Emergency department use, continuity of care.	28	Hospital care-coordination for homeless people; 14/17 studies reported fewer ED visits or readmissions (56.6–96.2 %, p = 0.013); 11/13 showed better housing stability (54.6–98.1 %, p = 0.023); all eight cost studies found savings (63.1–100 %, p = 0.008); continuity of care improved.	Moderate

**Table 7:** Summary of included reviews reporting digital and remote interventions

Study	Population	Intervention(s)	Outcomes	Number of primary studies	Summary of results	AMSTAR II quality appraisal
Western et al. 2023	Adults from socioeconomically disadvantaged backgrounds.	Digital physical activity and behavioural change interventions.	Physical activity, behavioural adherence, health literacy.	19	Digital physical-activity apps for low-SES adults; no within-group change, yet low-SES users showed a comparative benefit versus higher-SES peers (standardised estimate 0.34; 95 % CI 0.22 to 0.45); effect size unrelated to number of behaviour-change techniques, pointing to the value of context-specific tailoring.	Low
Katz et al. 2023	Adults with hypertension in low-income communities.	Telemonitoring and digital hypertension interventions.	Blood pressure, treatment adherence, patient activation.	28	Digital hypertension management in underserved adults; multi-component packages combining home BP monitors, mobile apps and clinician feedback lowered systolic BP, with the largest reductions in app-plus-provider integrations.	High
Obregon et al. 2024 [50]	Paediatric patients (0–18 years), from low-income families	eHealth	Wide variability in outcomes, due to study heterogeneity	9	Telehealth in paediatrics; generally acceptable and feasible for low-income families, though technology access and digital skills varied; few studies tracked downstream health outcomes, and none stratified results by SES.	Low
Egan et al. 2023	Adolescents in socioeconomically and geographically marginalised contexts	Digital public health interventions	Real-world behaviour change (e.g., smoking, diet)	15	Digital public-health tools for marginalised adolescents; social-media campaigns and app prompts yielded short-term drops in smoking and poor diet; effects inconsistent across SES, improving when interventions were paired with community infrastructure or supportive policy.	Moderate

**Table 8:** Summary of included reviews reporting educational, behavioural and psychosocial interventions

Study	Population	Intervention(s)	Outcomes	Number of primary studies	Summary of results	AMSTAR II quality appraisal
Troy et al. 2022 [31]	General adult population with low SES groups in educational settings.	Interventions within educational settings.	Aggression, academic engagement, health literacy	62	School-based programmes focusing on peer mentoring and behaviour modification showed improvements in aggression and academic engagement, but equity impacts were not consistently evaluated.	Moderate
Littlecott et al. 2022 [32]	Secondary school students in deprived areas	School-based peer networks	Behavioural problems, academic engagement	30	Health education initiatives in schools improved health literacy and behaviour in disadvantaged students, though subgroup analyses were limited.	Moderate
Van Heijster et al. 2021 [60]	Low socioeconomic populations in employment	Workplace-based health promotion	Self-perceived health	6	Workplace lifestyle programmes showed no change in self-perceived health for low-SEP workers (β 0.03; 95 % CI −0.03 to 0.09); no effect modification by gender, marital status or age.	Low
McDarby and Looney 2023	Parents of children with obesity	Group-based, parent-only obesity interventions	Child BMI, health behaviours	15	Parent-only obesity groups lowered child BMI and improved behaviours; treatment success by SES contradictory; income and education rarely analysed for attrition.	Critically Low
Orciari et al. 2022 [33]	Marginalised adults with housing instability or substance use	Motivational interviewing	Health engagement, behavioural change	11	Motivational interviewing for marginalised adults produced mixed results in engagement and behaviour.	Moderate
Hardman et al. 2020 [34]	Adults with long-term conditions	Self-management support	Clinical and behavioural outcomes	19	Generic self-management had lower uptake in low-income groups; SES-tailored versions produced significant clinical improvements.	Moderate
Mathiesen et al. 2019 [35]	Vulnerable individuals with type 2 diabetes	Psychosocial interventions	Diabetes-related distress, depression, HbA1c	18	Psychosocial support cut diabetes distress at 6 months (SMD −0.20) and 12 months (−0.21) and reduced depression (−0.20); no HR-QoL change; intensive individual formats slightly lowered HbA1c.	Low
Robinson et al. 2023 [36]	General adult population (mixed SES)	Menu energy labelling	Real-world calorie selection	17	Menu calorie labels produced no overall behavioural shift (SMD 0.094) and about 40 kcal fewer calories chosen; no SEP interaction.	High
Garbers et al. 2018 [37]	Ethnic minority men with low health literacy	Self-management support	Physiological (e.g., blood pressure, HbA1c), behavioural and psychosocial measures, illness knowledge, costs and service use	17	Multi-technique self-management for young, low-literacy minority men improved blood pressure, A1C and self-efficacy; effect sizes varied and tailoring was not always superior.	Critically Low
Feteira-Santos et al. 2020 [38]	General adult population with low SES inclusion	Front-of-pack nutritional labelling	Nutrition knowledge, food selection	9	Nutri-Score boosted healthy choices most in disadvantaged groups; traffic-light and HSR sometimes worsened choices for low-income men; two-colour comparative labels aided low-knowledge users.	Low
Shreshta et al. 2023	Adult low SES populations	Front-of-pack labelling (FOPL) on food	Product ranking; understanding of different FOPL formats	39	Review of front-of-pack nutrition labelling showed improved consumer comprehension and product selection. However, outcome differences by socioeconomic status were inconsistently reported, and stratified analysis was rare.	Moderate

### Evidence overlap between reviews

**Fig. 3:**
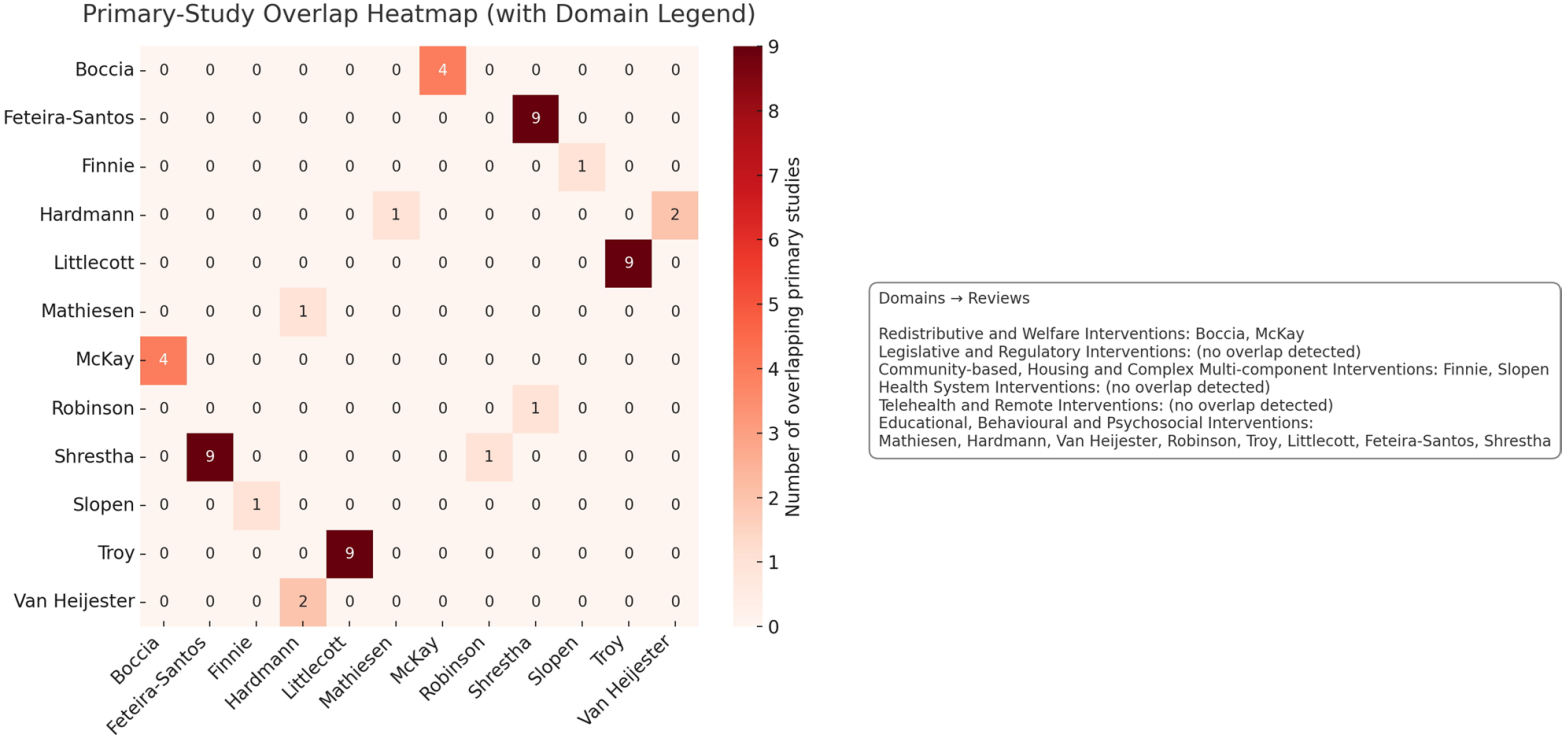
Heatmap displaying primary-study overlap across included systematic reviews, grouped by intervention domain

Minimal primary-study overlap was observed across the included reviews and was largely confined to reviews within the same intervention domain, as displayed in Fig. 3. Overlap was most evident within the *Redistributive and Welfare* and *Educational*,* Behavioural and Psychosocial* domains, while no duplication was found across the *Legislative*, *Health System*, or *Telehealth* domains. Overall, overlap accounted for less than ten per cent of all primary studies, indicating a broad and independent evidence base.

### Redistributive and welfare interventions

Six reviews (Table 3) assessed cash transfers, welfare schemes, basic income and food subsidy programmes across the life course, targeting children, families and low-income adults, with outcomes spanning service access, food security, financial stress, and psychosocial wellbeing [29, 30, 54, 57, 59, 63].

Cash transfer and income support schemes demonstrated modest yet consistent gains in intermediate outcomes. Fuller et al. [29] evidenced improvements in early childhood development, parental mental health and health-service uptake amongst low-income families. Boccia et al. [57] corroborated this with significantly higher neonatal birth weight under income support. McKay et al. [59] evaluated basic income models, including the Canadian MINCOME experiment and a Finnish trial: unconditional transfers to low-income individuals were associated with reduced hospitalisations, improved subjective wellbeing and lower stress; yet outcomes were not disaggregated by social group, leaving uncertain whether inequities narrowed or absolute outcomes rose uniformly. Gibson et al. [63], reviewing welfare-to-work programmes in low-income lone-parents, observed early gains in self-rated health and depression that attenuated over time.

McGrath et al. [54] examined place-based welfare for working-age adults facing financial insecurity: benefits were uneven. Co-located health and welfare services yielded small wellbeing improvements concentrated among recipients in receipt of financial support, and among women and Black participants. Link-worker social prescribing modestly reduced anxiety and service use; debt advice was largely ineffective. Active labour market policies reduced depression and improved functioning, chiefly among higher-educated jobseekers. Food banks improved food security and mental health, whereas empowerment-focused alternatives did not. Haslam et al. [30], synthesising 17 food-prescription studies among low-income adults with diet-related chronic disease, reported an additional 0.77 daily fruit-and-vegetable servings (95% CI 0.30 to 1.24) and a BMI reduction of 0.40 kg m⁻² (95% CI − 0.50 to − 0.31), with no significant change in HbA1c or systolic blood pressure.

### Legislative and regulatory interventions

Four reviews (Table 4) assessed legal and regulatory tools shaping exposure, access and affordability [47–49, 64].

Nanninga et al. found that public smoking bans reduced children’s exposure to second-hand smoke in the home (RR 0.72, 95% CI 0.62 to 0.83), with high heterogeneity (I² = 98.8%). Only one primary study conducted a subgroup analysis, indicating protection confined to higher-skilled occupation households [47]. Sanchez-Vaznaugh et al. [64] reported mixed results for US competitive food-and-beverage policies in schools: two Minnesota correlational studies associated stronger, more comprehensive policies with lower BMI percentiles in medium-poverty districts, with a significant benefit only in high-poverty schools; other studies showed no consistent differential by free-or-reduced-price-lunch status, gender, grade or race.

Kaplan et al. [48] reviewed legislative reforms to prescription drug access and affordability, including co-payment elimination, tiered subsidies and broader insurance expansion. Across 19 studies, reductions in out-of-pocket costs were consistently associated with improved adherence and pharmacy utilisation, predominantly among low-income and minoritised groups; differential effects were shaped by eligibility thresholds, administrative gatekeeping and co-pay design. Guindon et al. [49] examined public drug insurance and unmet need: coverage improved self-reported medication access and reduced cost-related non-adherence. Several studies reported narrowed income and education gradients in service use, although disparities persisted where health literacy or provider bias were unaddressed. Equity gains were greatest where legislative change was paired with outreach, simplification and embedded support; legal entitlement alone did not guarantee distributive equity.

### Community-based, housing and complex multi-component interventions

Nine reviews (Table 5) evaluated multi-modal programmes within community and housing contexts, frequently targeting structurally marginalised groups [39–44, 56].

Nickel and von dem Knesebeck [39] reported that 57% of 23 multicomponent neighbourhood interventions reduced inequity in health or related behaviours; one showed mixed or adverse effects, and two benefited only the most deprived. Four reviews focused on housing and the built environment. Onapa et al. [40] evidenced supportive housing for people experiencing homelessness with HIV/AIDS consistently improved survival and immune function, with fewer depressive symptoms and reduced suicidal ideation reported in some cohorts. Slopen et al. [56] demonstrated that housing support for low-income families yielded healthier weight-for-age and weight-for-height in children, fewer symptoms of psychological distress, and reductions in violent behaviour and substance abuse among voucher recipients; observational work corroborated gains in infant growth, iron status, asthma, outdoor play and behaviour. Finnie et al. [41], examining the US Housing Choice Voucher programme, found better housing and safer, lower-poverty neighbourhoods, small rises in employment, insurance coverage and food security, fewer adult physical and mental health problems, and reduced childhood asthma; long-term gains appeared for children receiving vouchers before age 13.

Specialised multicomponent interventions addressed clinically vulnerable groups. Ellis et al. [42] reported that tailored safer sleep programmes increased adherence to guidance, with higher uptake among low-income and ethnic minority families. Raison and Harris [43] found that antenatal oral health education and free dental packs increased service uptake among disadvantaged pregnant women. Anyiwe et al. [44] evidenced that educational outreach, culturally tailored messaging and patient navigation improved hepatitis B screening, knowledge and linkage to care among socially excluded populations, including migrants and people experiencing homelessness.

### Health system interventions

Three reviews (Table 6) examined interventions delivered within health-system settings, including primary care, hospital discharge coordination, specialist smoking cessation and clinical screening for social needs [45, 46, 55].

Allen et al. [55] reported that chronic-disease management, lifestyle support and health literacy initiatives improved patient activation, self-management and engagement among low-SES adults; few explicitly reduced inequalities. Smith et al. [45] found that tailored smoking cessation, frequently under culturally responsive models, improved quit rates among low-SES and disadvantaged groups, indicating potential to narrow tobacco-related gradients. Luchenski et al. [46] synthesised hospital-based care coordination for people experiencing homelessness: across sixteen weak, ten moderate and two high-quality RCTs, 14 of 17 evaluations recorded fewer emergency visits or readmissions (56.6 to 96.2%, *p* = 0.013), all eight cost studies reported savings (63.1 to 100%, *p* = 0.008), and 11 of 13 observed improved housing stability (54.6 to 98.1%, *p* = 0.023); continuity of care and post-discharge follow-up also improved.

### Telehealth and remote interventions

Four reviews (Table 7) evaluated digital and telehealth approaches among disadvantaged populations, spanning mobile applications, remote monitoring, eHealth platforms and hybrid models [50, 52, 53, 58].

Western et al. [53] reported no significant within-group gains for low-SES participants in digital physical-activity interventions; however, a comparative analysis favoured low SES when disaggregated (standardised estimate 0.34, 95% CI 0.22 to 0.45). The number of behaviour-change techniques did not correlate with effect size, underscoring the salience of context, design specificity and delivery tailoring. Egan et al. [52] examined adolescent-facing digital public health in socioeconomically and geographically marginalised settings, including social-media campaigns, app-based prompts and curriculum supplements: several studies showed short-term reductions in risk behaviours such as smoking and poor diet, with effects stronger where interventions were co-delivered with community infrastructure or policy support and variable across SES. Katz et al. [58] found that multi-component digital hypertension management improved systolic blood pressure in underserved populations, with the largest gains under app-based self-monitoring with clinician oversight. Obregon et al. [50] reported paediatric telehealth to be acceptable and feasible among low-income families; access and digital skill varied, and few studies measured downstream clinical outcomes.

### Educational, behavioural and psychosocial interventions

Eleven reviews (Table 8) covered interventions delivered through schools, workplaces, parental settings and population-facing educational or behavioural platforms, seeking to modify behaviours, enhance health literacy and promote psychosocial wellbeing, with outcomes spanning diet, mental health, school performance and chronic-disease self-management [31–38, 60–62].

School-based programmes investigated by Troy et al. [31] and Littlecott et al. [32], encompassing peer mentoring, health education and behavioural skill-building, reduced aggression, improved academic engagement and health literacy; effects were socially patterned, with the most deprived pupils benefiting least and larger effect sizes in better resourced schools. Mental health outcomes were mixed among disadvantaged children. Van Heijster et al. [60], using individual participant data meta-analysis, reported no improvement in self-perceived health among low-socioeconomic-position workers (β = 0.03, 95% CI − 0.03 to 0.09); significant gains accrued to higher-SES workers. Overall, multicomponent workplace lifestyle programmes did not narrow inequality and may, in certain contexts, widen inequality.

Parental programmes reviewed by McDarby and Looney [62] evidenced group-based, parent-only obesity interventions improved child BMI and health behaviours; findings for low-SES groups were contradictory. Orciari et al. [33] found motivational interviewing among marginalised populations with housing instability or substance use yielded mixed effects on engagement and behaviour.

Three reviews addressed behavioural and psychosocial self-management. Hardman et al. [34] demonstrated lower enrolment and completion among low-income groups; however, where programmes were tailored to low SES, significant clinical improvements in chronic conditions followed. Mathiesen et al. [35] identified small but significant reductions in diabetes distress at 6 months (SMD − 0.20, 95% CI − 0.31 to − 0.08, moderate evidence) and 12 months (SMD − 0.21, − 0.34 to − 0.09); depression also decreased (four studies, SMD − 0.20, − 0.33 to − 0.07, low evidence). Intensive, individual formats produced larger distress reductions than brief or group formats; only intensive programmes slightly lowered HbA1c (MD − 0.23, − 0.44 to − 0.01). Garbers et al. [37] reported that multi-technique self-management for young, ethnic minority men with low health literacy or low-income improved blood pressure, A1C and self-efficacy, although effect magnitude varied and tailored components did not invariably outperform standard approaches.

Large-scale behavioural interventions included menu and front-of-pack labelling. Robinson et al. [36] showed menu energy labelling reduced calories chosen by about 40 kcal (95% CI − 75 to − 5, *p* = 0.025), without socioeconomic differentials. Feteira-Santos et al. [38] reported that interpretive front-of-pack systems, notably Nutri-Score, generated healthier perceptions and choices, with the largest gains among low-SES consumers; simpler two-colour formats aided those with low educational attainment. More complex schemes sometimes worsened choices among low-income men. Shrestha et al. [61] extended this evidence base, demonstrating enhanced capacity among low-SES consumers to identify healthier options. Overall, labelling modestly reduced inequity in healthy choice and caloric intake; presentation and modality were decisive for equity.

## Discussion

### Evolution of the evidence

Our umbrella review serves as a high-quality update from the previous methodologically rigorous umbrella review by Thomson et al. from 2018 [18]; which in turn, functioned as a partial update from the 2013 umbrella review by Lorenc et al. (2014) [19], and the seminal work by Bambra et al. (2010) [20]. Together, these reviews established that structural, population-level policies tend to yield the greatest equity gains, while highlighting persistent gaps in evaluative consistency. Naik et al. (2019) [65] later examined macroeconomic determinants of health and inequality across all income settings; however, the review’s global scope and narrower focus solely on upstream fiscal and labour-market contexts differed from the wider public-health and social policy scope of Thomson et al. Our review therefore continues directly from Thomson, maintaining a high-income country focus and comparable inclusion parameters, while drawing contextual insight from the broader findings of Naik et al. By incorporating 35 systematic reviews conducted in high-income countries, this review represents the most comprehensive update of the evidence base since 2018. This review extends prior work through inclusion of novel intervention domains, while applying the Health Equity Pyramid conceptual framework to interpret agentic demand and population reach. To the best of our knowledge, no other umbrella review on this subject area has focused exclusively on high-income countries in the intervening period. While OECD membership has undergone minor expansion since 2018, these changes are not relevant to this review’s inclusion scope, and country classification remained consistent with current OECD listings. This review’s distinct contribution lies not in novelty of the topic but in scope, recency, and the conceptual application of agentic demand via the Health Equity Pyramid framework. Thereby, developing six themes with stepwise gradation of individual agency against population impact: (1) redistributive and welfare interventions; (2) Educational, behavioural and psychosocial programmes; (3) Community-based, housing and complex multicomponent model; (4) Health-system interventions; (5) Legislative and regulatory measures; (6) Telehealth and remote digital interventions.

### Efficaciousness of public health policies on health inequity

Employing our conceptual framework of the health equity pyramid (Fig. 1), we assessed the included evidence on an agentic demand scale: with the proposition that public health policies requiring less agentic demand sustain more population impact and reach: with the reverse also being true. The emergence of this dichotomy within public health is not a new phenomenon, being discussed first in 1985 [66]; yet only in comparative recency being deployed in the assessment of public health intervention efficacy. Within the context of this review, we define individual agency as: “*the extent of personal resources—cognitive*,* material and time—required from each person to benefit from an intervention”* [67]. Interventions characterised by low agentic demand require minimal individual effort or decision-making, often operating through structural or contextual change at the population level; whereas those with high agentic demand rely on sustained personal engagement, motivation, and capacity to achieve benefit [68]. Below are the three themes to emerge when applying the conceptual framework to the included evidence base:

### Low agentic demand; greater population impact

The intervention domains included in this review which evidence low agentic demand whilst having the greatest population reach and impact are redistributive and welfare policies; legislative and regulatory interventions; and certain community-based, housing and complex multi-component interventions. These interventions are epitomised by changing population context and socioeconomic factors through modulating the fiscal, physical or regulatory environment. The included systematic reviews from the aforementioned domains demonstrate robust evidence on reducing health inequity in low-SES populations. These policies mitigate cognitive effort and individual action; therefore reducing decision-making burden in low-SES populations by making a health-generating choice the default option [69]. Therefore, emphasising the need for public health policies that interact with a complexity of multiple determinants to reshape context for durable equity gains [70]. Within the wider literature, low agentic demand studies have yielded sizable population health gains, with examples including mandatory fortification of routine foods [71], taxation of hazardous products [72], and smoke-free legislation [73].

### High agentic demand; lower population impact

The included systematic reviews which demanded greater agentic demand included certain telehealth interventions; educational, behavioural and psychosocial interventions; and clinical interventions. Due to these public health interventions requiring greater cognitive demand and decision-marking burden; naturally, these interventions exploit greater health literacy, time availability, motivation and economic resources [74]; possibly resulting in inequitable intervention uptake and participation. Within the context of this umbrella review, high agentic demand public health policies produced mixed results: interventions which were specifically targeted towards low-SES populations, taking into account educational status and cultural sensitivity, were generally favourable. The converse was true for interventions without contextualisation. Specifically, interventions which reduced cognitive burden or targeted barriers in specific high-risk populations, were efficacious in mitigating health inequity in contrast to interventions neglecting these factors. This is corroborated by prior research demonstrating the success of high agency interventions to be dependent on simultaneously reducing cognitive, material and time costs for low-SES groups [74, 75]. Whilst acknowledging the lower population impact of high agentic public health policies, this review evidenced efficaciousness of high agentic interventions in select populations. Clinical interventions, embedded in the health system infrastructure, can achieve equity gains when precisely targeted to specific disadvantaged populations [76], particularly when risk is highly concentrated due to both disease prevalence and low-SES [77].

### Intervention-generated inequality (IGI)

High agentic demand interventions, without being tailored to low-SES or disadvantaged populations, may lead to intervention-generated inequality; whereby, a public health policy, while possibly improving overall population health, exacerbates socioeconomic inequality through disproportionately benefitting more affluent groups than less affluent groups [19]. Our review demonstrated IGI, confined to high-agentic demand public health interventions, with relatively low population impact. Specifically, Feteira-Santos et al. [38], evidenced low-income men choosing less nutritious products than higher-income men during a front-of-pack nutritional labelling intervention trial; Littlecott et al. [32], exploring a school peer networking intervention, also demonstrated worse behavioural outcomes in low-SES pupils, and improved school resource and affluence to be a key indicator of behavioural outcome; Hardman et al. [34], demonstrated clinical benefits accrued chiefly to higher-literacy groups when the intervention was not explicitly tailored. Social determinants are critical modulators of health behaviour. Interventions that impose high agentic demands without addressing these underlying structures produced intervention-generated inequalities, within our review, conferring benefit primarily on those already equipped with material and cognitive resources; benefitting low-SES groups the least [78, 79]. Notably, IGI was exclusive to high agentic public health policies; however, the majority of included systematic reviews of high-agentic interventions did not produce IGI.

### Strengths

This PROSPERO registered review employed methodological rigour with a robust and comprehensive search strategy which identified comprehensive evidence on public health policies and interventions to address health inequity. This review utilised the Joana Briggs Institute (JBI) guidelines and Cochrane handbook to adhere to the highest standards of umbrella review methodology [80, 81]: with the JBI emerging as gold-standard guidance in umbrella review methodology [81]. Additionally, this review followed the PRIOR statement checklist to ensure standardised reporting [82]. The study also enhanced methodological breadth by searching the pertinent medical databases. This review included comprehensive evidence on the subject, with less than ten per cent overlap of primary studies between the included reviews. Furthermore, we included a broad range of interventions at the national or state-level; this enhanced population reach of the interventions.

### Limitations

Our umbrella review is subject to certain limitations. Firstly, due to the inclusion of a multiplicity of interventions and public health policies, our included systematic reviews are subject to significant heterogeneity due to difference in intervention type within each domain; and consequently, differing intra- and inter-domain outcome measurements. Secondly, the primary evidence base in the included systematic reviews is dominated by non-randomised controlled trials; this predominance is a natural consequence of public-health research and leaves residual confounding inadequately addressed. Thirdly, a consequence of the umbrella review study design, is the possibility of primary study overlap between systematic reviews, leading to the possibility of double-counting within the evidence base. The use of the contemporaneous AMSTAR-II risk of bias tool, only ranking three studies as high-confidence, raises the possibility of under-estimation of confidence in complex, quasi-experimental study designs, overrepresented in interventional public health research, through stringent criteria [83] and lack of emphasis on context [84]. Nonetheless, this focus is beyond the scope of this review. Of note, no eligible systematic reviews examined the impact of public health policy on violence against children in high-income countries, with a focus on explicit health or health-related outcome measures. The absence of policy-level evaluations therefore constitutes an inherent gap in the underlying literature and constrains inferences in this area.

### Policy and practice implications

For stakeholders and policymakers, this review underscores the imperative of prioritising public health policies that alter population-level contexts and structures, thereby minimising the individual burden of health-related decision-making among disadvantaged groups. This review highlights promising interventions for policymakers to consider implementing within high-income countries, which are low-agentic demand, yet produce greater population impact. Specifically, welfare system reform, including conditional and unconditional cash transfer programmes, universal basic income models, and healthy food subsidies; legislative and regulatory reform, particularly in relation to healthcare system reform by reducing out-of-pocket drug costs and expenditure; and community-based interventions, with housing interventions chiefly mitigating health inequity. This review does not preclude the use of high individual agency interventions; but urges tailoring to cultural and socioeconomic context of disadvantaged populations. Policymakers should note that, in clinical contexts, delivered to specific groups, with disadvantage mediated by both disease process and low-SES status, such interventions can yield significant clinical benefit and parity.

## Conclusion

Our umbrella review extends previous research into the efficacy of public health policy in addressing health inequity in high-income countries. The evidence presented in the review clearly indicates that public health policies, characterised by low agentic demand, such as redistributive welfare measures, robust legislative regulations, and community and housing support programmes, consistently yield robust reductions in health inequities, predominantly benefiting disadvantaged populations. Conversely, interventions necessitating higher agentic demand via cognitive, motivational, and resource commitments from individuals yielded poorer outcomes for disadvantaged populations; and critically, in some instances, led to intervention-generated inequalities by disproportionately favouring higher socioeconomic groups. However, methodological challenges intrinsic to umbrella reviews must be taken into consideration in the interpretation of these findings. Additionally, further methodological refinement and validation of appraisal instruments suited explicitly to complex, real-world public health interventions are critical. Future research should prioritise addressing existing gaps identified, particularly through rigorous equity-focused evaluations. Ultimately, achieving equitable and sustainable health outcomes demands a policy landscape that prioritises structural interventions across the social determinants of health.

## Supplementary Information

Supplementary Material 1.

Supplementary Material 2.

Supplementary Material 3.

## Data Availability

All data generated or analysed during this study are included in this published article (and its additional files). The extraction forms for all the individual systematic reviews are available from the corresponding author on reasonable request.

## Abbreviations

AMSTAR: A Measurement Tool to assess systematic reviews. Used to evaluate the quality of systematic reviews 2
PICOS: Population, Intervention, Comparison, Outcomes, Setting. Framework used for eligibility criteria
PRISMA: Preferred Reporting Items of Systematic Reviews and Meta-Analyses
PROGRESS-Plus: Acronym identifying equity dimensions (e.g. socioeconomic status, place of residence, ethnicity, gender). Used to map populations and outcomes
AIDS: Acquired Immune Deficiency Syndrome. Used in the context of housing interventions for people experiencing homelessness with HIV/AIDS
HIV: Human Immunodeficiency Virus. Used in relation to supportive housing interventions improving survival and outcomes
BMI: Body Mass Index. Used as an outcome measure in food subsidy and parental obesity interventions
COVID-19: Coronavirus Disease. Cited in relation to widening inequities during the pandemic
DARE: Database of Abstracts of Reviews of Effects. Used as the benchmark for minimum quality standards for systematic reviews
IGI: Intervention-Generated Inequality. Describes situations where high agentic interventions exacerbate socioeconomic disparities
JBI: Joanna Briggs Institute. Used to signal methodological guidelines underpinning the umbrella review
MINCOME: Manitoba Basic Annual Income Experiment. Referenced in basic income trials assessing welfare interventions
OECD: Organisation for Economic Co-operation and Development. Defines eligible high-income countries in the review
PROSPERO: International Prospective Register of Systematic Reviews. Registration platform for this umbrella review
SES: Socioeconomic Status. Primary axis for health inequity in included studies
SMD: Standardised Mean Difference. Statistical effect measure used in meta-analyses of psychosocial and chronic disease interventions
