# Supplementary material for: Public health policies and interventions to address health inequities in high-income countries: an umbrella review
Source: BMC Public Health. 2026 Feb 5;26:473. doi: 10.1186/s12889-025-25876-2 (PMC12874971; doi:10.1186/s12889-025-25876-2)
Supplement: Supplementary file 2 — Supplementary Material 2. [file 12889_2025_25876_MOESM2_ESM.pdf]

Search Name:
Date Run: 23/09/2024 18:19:37
Comment:

ID Search Hits
#1 ("educational status" OR (education* NEAR/2 level*) OR ((higher OR better OR worse OR less) NEAR/1 educated) OR ((higher OR better OR worse OR less) NEAR/1 level* AND of AND education))
11136
#2 (disparit* OR inequalit* OR inequit* OR equity OR deprivation OR socio-economic OR socioeconomic OR ses OR impoverished OR poverty OR "economic level") 34143
#3 ("health disparit*" OR "health inequalit*" OR "health inequit*" OR "medically underserved") 609
#4 #1 OR #2 OR #3 43598
#5 ("potential determinants" OR "significant correlates of" OR "variables associated with" OR "determinants of" OR "factors associated with" OR "identif* determinants" OR "risk factors for" OR ("significantly related to" OR "significant predictor") OR (also NEAR/2 "associated with") OR ("at increased risk" OR "at decreased risk") OR "association* between" OR ("positively associated" OR "negatively associated") OR "differed by" OR ("were high* amongst" OR "were low* amongst") OR ("inverse relationship with" OR "inversely associated with" OR "inversely related to") OR "reverse association" OR "differentially affects" OR "evidence of a link between" OR (significantly NEAR/3 "likelihood of") OR "protective factors for" OR (differ* NEAR/2 "according to") OR (inverse NEAR/2 gradient) OR (positive NEAR/2 gradient) OR (negative NEAR/2 gradient) OR ("trends were" NEAR/3 across) OR ("related to" NEAR/3 variable*) OR ("differences were" NEAR/3 "explained by"))
102985
#6 (intervention* OR polic*) 656712
#7 ((systematic NEAR/1 review*) OR meta-analys* OR metaanalys*)
39844
#8 #4 and #5 and #6 and #7 with Cochrane Library publication date Between May 2017 and Aug 2024, in Cochrane Reviews 837
#9 (afghanistan OR albania OR algeria OR "american samoa" OR angola OR "antigua and barbuda" OR antigua OR barbuda OR argentina OR armenia OR armenian OR aruba OR azerbaijan OR bahrain OR bangladesh OR barbados OR "republic of belarus" OR belarus OR byelarus OR belorussia OR byelorussian OR belize OR "british honduras" OR benin OR dahomey OR bhutan OR bolivia OR "bosnia and herzegovina" OR bosnia OR herzegovina OR botswana OR bechuanaland OR brazil OR brasil OR bulgaria OR "burkina faso" OR "burkina fasso" OR "upper volta" OR burundi OR urundi OR "cabo verde" OR "cape verde" OR cambodia OR kampuchea OR "khmer republic" OR cameroon OR cameron OR cameroun OR "central african republic" OR "ubangi shari" OR chad OR chile OR china OR colombia OR comoros OR "comoro islands" OR "iles comores" OR mayotte OR "democratic republic of the congo" OR "democratic republic congo" OR congo OR zaire OR "costa rica" OR "cote divoire" OR "cote d ivoire" OR "cote divoire" OR "cote d ivoire" OR "ivory coast" OR croatia OR cuba OR cyprus OR "czech republic" OR czechoslovakia OR djibouti OR "french somaliland" OR dominica OR "dominican republic" OR ecuador OR egypt OR "united arab

republic" OR "el salvador" OR "equatorial guinea" OR "spanish guinea" OR eritrea OR estonia OR eswatini OR swaziland OR ethiopia OR fiji OR gabon OR "gabonese republic" OR gambia OR "georgia (republic)" OR georgian OR ghana OR "gold coast" OR gibraltar OR greece OR grenada OR guam OR guatemala OR guinea OR "guinea bissau" OR guyana OR "british guiana" OR haiti OR hispaniola OR honduras OR hungary OR india OR indonesia OR timor OR iran OR iraq OR "isle of man" OR jamaica OR jordan OR kazakhstan OR kazakh OR kenya OR "democratic peoples republic of korea" OR "republic of korea" OR "north korea" OR "south korea" OR korea OR kosovo OR kyrgyzstan OR kirghizia OR kirgizstan OR "kyrgyz republic" OR kirghiz OR laos OR "lao pdr" OR "lao people's democratic republic" OR latvia OR lebanon OR "lebanese republic" OR lesotho OR basutoland OR liberia OR libya OR "libyan arab jamahiriya" OR lithuania OR macau OR macao OR "macedonia (republic)" OR macedonia OR madagascar OR "malagasy republic" OR malawi OR nyasaland OR malaysia OR "malay federation" OR "malaya federation" OR maldives OR "indian ocean islands" OR "indian ocean" OR mali OR malta OR micronesia OR "federated states of micronesia" OR kiribati OR "marshall islands" OR nauru OR "northern mariana islands" OR palau OR tuvalu OR mauritania OR mauritius OR mexico OR moldova OR moldovian OR mongolia OR montenegro OR "montenegro (republic)" OR morocco OR ifni OR mozambique OR "portuguese east africa" OR myanmar OR burma OR namibia OR nepal OR "netherlands antilles" OR nicaragua OR niger OR nigeria OR oman OR muscat OR pakistan OR panama OR "papua new guinea" OR "new guinea" OR paraguay OR peru OR philippines OR philipines OR phillipines OR phillippines OR poland OR "polish people's republic" OR portugal OR "portuguese republic" OR puerto rico OR romania OR russia OR "russian federation" OR ussr OR "soviet union" OR "union of soviet socialist republics" OR rwanda OR ruanda OR samoa OR "pacific islands" OR polynesia OR "samoan islands" OR "navigator island" OR "navigator islands" OR "sao tome and principe" OR "saudi arabia" OR senegal OR serbia OR seychelles OR "sierra leone" OR slovakia OR "slovak republic" OR slovenia OR melanesia OR "solomon island" OR "solomon islands" OR "norfolk island" OR "norfolk islands" OR somalia OR "south africa" OR "south sudan" OR "sri lanka" OR ceylon OR "saint kitts and nevis" OR "st. kitts and nevis" OR "saint lucia" OR "st. lucia" OR "saint vincent and the grenadines" OR "saint vincent" OR "st. vincent" OR grenadines OR sudan OR suriname OR surinam OR "dutch guiana" OR "netherlands guiana" OR syria OR "syrian arab republic" OR tajikistan OR tadjikistan OR tadzhikistan OR tadzhik OR tanzania OR tanganyika OR thailand OR siam OR "timor leste" OR "east timor" OR togo OR "togolese republic" OR tonga OR "trinidad and tobago" OR trinidad OR tobago OR tunisia OR turkey OR "turkey (republic)" OR turkmenistan OR turkmen OR uganda OR ukraine OR uruguay OR uzbekistan OR uzbek OR vanuatu OR "new hebrides" OR venezuela OR vietnam OR "viet nam" OR "middle east" OR "west bank" OR gaza OR palestine OR yemen OR yugoslavia OR zambia OR zimbabwe OR "northern rhodesia" OR "global south" OR "africa south of the sahara" OR "sub saharan africa" OR "subsaharan africa" OR "africa, central" OR "central africa" OR "africa, northern" OR "north africa" OR "northern africa" OR magreb OR maghrib OR sahara OR "africa, southern" OR "southern africa" OR "africa, eastern" OR "east africa" OR "eastern africa" OR "africa,

western" OR "west africa" OR "western africa" OR "west indies" OR "indian ocean islands" OR "caribbean region" OR "caribbean islands" OR caribbean OR "central america" OR "latin america" OR "south and central america" OR "south america" OR "asia, central" OR "central asia" OR "asia, northern" OR "north asia" OR "northern asia" OR "asia, southeastern" OR "southeastern asia" OR "south eastern asia" OR "southeast asia" OR "south east asia" OR "asia, western" OR "western asia" OR "europe, eastern" OR "east europe" OR "eastern europe" OR "developing country" OR "developing countries" OR "developing nation?" OR "developing population?" OR "developing world" OR "less developed countr*" OR "less developed nation?" OR "less developed population?" OR "less developed world" OR "lesser developed countr*" OR "lesser developed nation?" OR "lesser developed population?" OR "lesser developed world" OR "under developed countr*" OR "under developed nation?" OR "under developed population?" OR "under developed world" OR "underdeveloped countr*" OR "underdeveloped nation?" OR "underdeveloped population?" OR "underdeveloped world" OR "middle income countr*" OR "middle income nation?" OR "middle income population?" OR "low income countr*" OR "low income nation?" OR "low income population?" OR "lower income countr*" OR "lower income nation?" OR "lower income population?" OR "underserved countr*" OR "underserved nation?" OR "underserved population?" OR "underserved world" OR "under served countr*" OR "under served nation?" OR "under served population?" OR "under served world" OR "deprived countr*" OR "deprived nation?" OR "deprived population?" OR "deprived world" OR "poor countr*" OR "poor nation?" OR "poor population?" OR "poor world" OR "poorer countr*" OR "poorer nation?" OR "poorer population?" OR "poorer world" OR "developing econom*" OR "less developed econom*" OR "lesser developed econom*" OR "under developed econom*" OR "underdeveloped econom*" OR "middle income econom*" OR "low income econom*" OR "lower income econom*" OR "low gdp" OR "low gnp" OR "low gross domestic" OR "low gross national" OR "lower gdp" OR "lower gnp" OR "lower gross domestic" OR "lower gross national" OR lmic OR lmics OR "third world" OR "lami countr*" OR "transitional countr*" OR "emerging economies" OR "emerging nation*") 336485

#10   #8 NOT #9   97
